# Climate stability and societal decline on the margins of the Byzantine empire in the Negev Desert

**DOI:** 10.1038/s41598-020-58360-5

**Published:** 2020-01-30

**Authors:** Petra Vaiglova, Gideon Hartman, Nimrod Marom, Avner Ayalon, Miryam Bar-Matthews, Tami Zilberman, Gal Yasur, Michael Buckley, Rachel Bernstein, Yotam Tepper, Lior Weissbrod, Tali Erickson-Gini, Guy Bar-Oz

**Affiliations:** 1 Department of Anthropology, Washington University in St Louis, 1 Brookings Dr., St Louis, 63130 MO USA; 2 Zinman Institute of Archaeology, University of Haifa, 199 Aba-Hushi Avenue, Haifa, 3498838 Israel; 3 Department of Anthropology, University of Connecticut, 354 Mansfield Road, Storrs, CT 06269 USA; 4 Department of Geochemistry, Geological Survey of Israel, 32 Yesha’ayahu, Leibowits Str., Jerusalem, 9692100 Israel; 5 Department of Earth and Environmental Sciences, Manchester Institute of Biotechnology, University of Manchester, Manchester, M13 9PL UK; 6 Jacob Blaustein Institutes for Desert Research, Ben-Gurion University of the Negev 653, Be’er Sheva, Israel; 7 Archaeological Division, Israeli Antiquities Authority, Tel Aviv, 61012 Israel

**Keywords:** Biogeochemistry, Environmental social sciences

## Abstract

Understanding past human settlement of inhospitable regions is one of the most intriguing puzzles in archaeological research, with implications for more sustainable use of marginal regions today. During the Byzantine period in the 4^th^ century CE, large settlements were established in the arid region of the Negev Desert, Israel, but it remains unclear why it did so, and why the settlements were abandoned three centuries later. Previous theories proposed that the Negev was a “green desert” in the early 1^st^ millennium CE, and that the Byzantine Empire withdrew from this region due to a dramatic climatic downturn. In the absence of a local climate archive correlated to the Byzantine/Early Islamic transition, testing this theory has proven challenging. We use stable isotopic indicators of animal dietary and mobility patterns to assess the extent of the vegetative cover in the desert. By doing so, we aim to detect possible climatic fluctuations that may have led to the abandonment of the Byzantine settlements. The findings show that the Negev Desert was not greener during the time period under investigation than it is today and that the composition of the animals’ diets, as well as their grazing mobility patterns, remained unchanged through the Byzantine/Early Islamic transition. Favoring a non-climatic explanation, we propose instead that the abandonment of the Negev Byzantine settlements was motivated by restructuring of the Empire’s territorial priorities.

## Introduction

Over the last 12,000 years, the Negev Desert in southern Israel has hosted an arid to hyper-arid climate, but despite the harsh living conditions, people have periodically established settlements here that have persisted for centuries^1,2^. Ongoing debate has seen archaeologists, historians, and climate scientists speculate whether the rise and fall of human habitation was caused by fluctuations in climatic conditions, or whether it was primarily driven by changes in the socio-political organization of the communities living in these environmentally marginal areas^3–7^. Palaeoenvironmental studies have contributed to this debate by providing a long-term perspective on climate fluctuations in the region. However, in order to understand the human response to these patterns on short-term (i.e. centennial) scales and ground the trends in specific micro-regional settings, archaeological proxies need to be studied from contexts that bracket the cultural events under consideration^8^.

One of the most significant phases of population expansion in the Negev Desert started in the 3^rd^ century BCE with the establishment of the Nabatean kingdom. After the kingdom was annexed and subsumed into the Roman empire in 106 CE, it reached its height with the spread of Christianity during the Byzantine period in the 4^th^–6^th^ century CE and was subsequently abandoned around the time of the Muslim conquest of the region in the 7^th^ century CE^9,10^. Between the 4^th^–7^th^ centuries CE, five major Byzantine settlements – Elusa (Halutza), Subeita (Shivta), Nessana (Nitzana), Avdat (Oboda), and Mamshit (Mampsis) – flourished in the Central Negev Desert, with several smaller pastoral farms spread around the landscape^11,12^. The inhabitants of these sites established a sophisticated system of water management that enabled them to capture runoff water for agricultural as well as domestic purposes. Approximately 30,000 acres of terraced fields were created in the hinterlands of the settlements, which made this phase of human occupation possible^6^. However, it is unclear why the systems declined within three centuries.

In this study, we use dietary proxies of domestic ovicaprids (sheep and goats) recently excavated at the Byzantine/Early Islamic town of Subeita (Shivta) and Nessana (Nitzana) and the Byzantine city of Elusa (Halutza)^13^. Although a mix of ancient Greek and Hebrew names, we use the names Shivta, Nessana and Elusa to make the terminology consistent with previous publications of these archaeological assemblages. The aim of the study is to 1) better understand the environmental conditions in which the Byzantine settlements developed, and 2) assess whether any climatic shifts may have led to the end of Byzantine presence in the Negev Desert. Stable carbon (𝛿^13^C, ratio of ^13^C/^12^C) and stable oxygen (δ^18^O, ratio of ^18^O/^16^O) isotopic values of tooth enamel carbonate, and stable carbon and nitrogen (δ^15^N, ratio of ^15^N/^14^N) isotopic values of tooth dentine and mandibular bone collagen are used to assess the grazing behaviour and mobility patterns of the animals. These reflect the type of vegetation and thus the climatic opportunities in the settled parts of the desert.

### Climate history of the southern Levant

**Figure 1:**
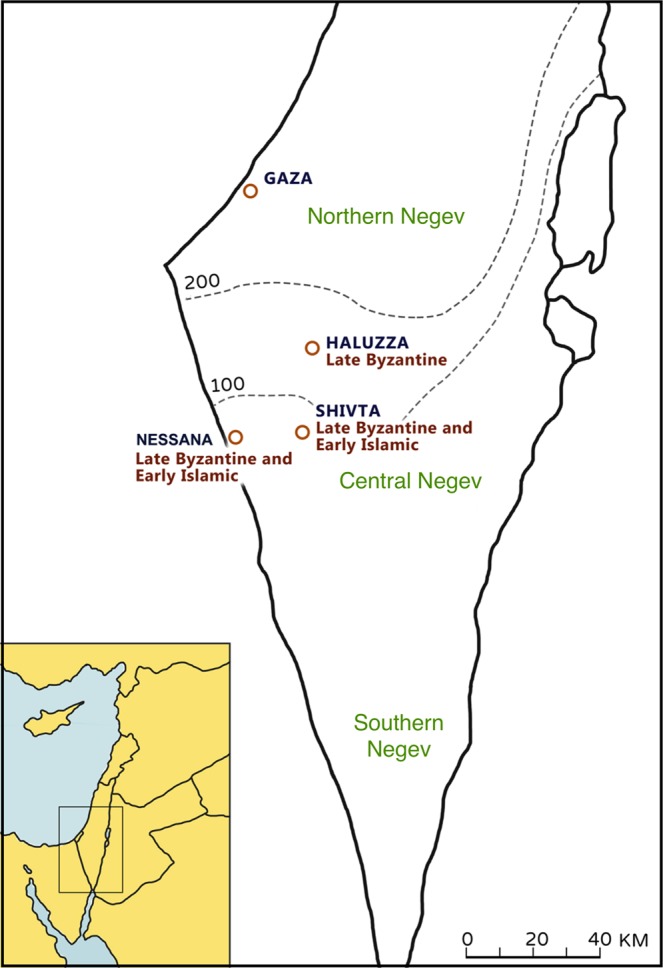
Location of study sites in the Negev Desert, southern Levant. Elusa (Halutza), Nessana (Nitzana) and Subeita (Shivta). Adapted from Marom *et al*.^13^.

The Negev Desert is located in the northern tropical global desert belt (30°N) and occupies a triangular region between the Mediterranean coast of southern Levant 50 km west of the modern city of Be’er Sheva, the Gulf of Aqaba and the southern tip of the Dead Sea (See Fig. 1). The region hosts a varied topography reaching altitudes of 1000 m.a.s.l. in the Southern Negev highlands, and below sea level in the Northern Arava Valley. The climate is arid in the north, with mean annual rainfall averaging 100–150 mm/yr (Aridity index ≤0.10), and hyper-arid in the regions of the Central Negev highlands (mean annual rainfall of 80–100 mm/yr) and the south (mean annual rainfall of less than 50 mm/yr) (Aridity index for both regions ≤0.06). During the wet season (October–April), vegetation is primarily concentrated in drainage channels, or *wadis*, that channel rainfall northwestwards towards the Mediterranean Sea or northeastwards towards the Dead Sea^14^. When runoff water is added to the rainfall, the amount of water in the *wadis* on an average year can exceed 200–300 mm/yr^14,15^. The loess soil above the dams in the *wadis* can then preserve moisture through the long and dry summers^3^.

Scholars attempting to understand the scale of the population expansion in the Negev desert during Antiquity have drawn on documented climate fluctuations across the southern Levant to argue that favorable climatic conditions made the settlement of the desert region more manageable than today. Horowitz^16^ observed that two Early Holocene pollen sequences from the Negev Desert were composed of a more diverse assemblage of plant taxa compared to a modern pollen sequence and concluded that Negev vegetation during the Early Holocene was more extensive and varied compared to the present day. Goodfriend^17^ used carbon isotopic measurements of land snails from the Negev Desert to argue that a higher proportion of the more water-demanding C_3_ vegetation reflected in the snail diets indicates that the region was wetter between 2800–4000 years BP compared to today. In their analysis of Dead Sea lake level changes during the Holocene, Enzel *et al*.^18^ proposed that the temporarily wetter phases documented by Goodfriend were restricted to the region of the northern Negev. The southern Negev – which has gone through its own climate history – has been arid continuously since the Late Pleistocene, as evident from the low formation of hyper-arid soils^19^.

Stable carbon and oxygen isotope values of the speleothem archive from the Soreq Cave in the Judean Mountains^20^ and comparison of this land archive to the marine stable oxygen isotope archive from the Eastern Mediterranean^21,22^ suggests that the climate in the Levant was wetter than today in the period prior to 7000 years BP. Between 7000–500 BP, it experienced a gradual decrease in precipitation and increase in temperature which eventually lead to aridification. Orland *et al*.^23^ analyzed the Soreq speleothem sequence using high-resolution ion microprobe seasonal sampling and extrapolated that rainfall decreased by ~200 mm/yr between 100–700 CE. Bookman *et al*.^24^ obtained a high-resolution sequence for the lake level changes in the Dead Sea and concluded that significant periods of ‘high-stand’ of the water level coincided with the Roman period in the 1^st^-2^nd^ centuries CE, the Byzantine period peaking in the 4^th^ century CE and the Crusader period in the 10–12th century CE. The periods of ‘high-stand’ were contrasted by a significant phase of ‘low-stand’, which started in the 5^th^ century CE. Both Bookman and Orland *et al*. argue that the climatic shifts leading to the decreased precipitation and low lake levels in the Dead Sea in the mid-1^st^ millennium CE were responsible for the decline of the Byzantine settlements, facilitating the eventual Muslim conquest. However, a decrease of 200 mm/yr in the Judean Mountains, and fluctuations in the Dead Sea levels, do not directly inform us what happened to the rainfall amounts in the Negev Desert to the south.

Avni *et al*.^14^ studied the geomorphological history of *wadis* in the Negev Desert, and argue that increased runoff and sedimentation of loess soils starting in the mid-Holocene made the environment naturally arable. Referring to it as the Desert Agriculture Geomorphic Window, the authors propose that it was these shifts in geomorphic conditions (i.e., changes in the sedimentation and erosion rates) that made the desert agriculture possible in Antiquity. These formation processes have now been reversed and in recent years, the *wadis* have been undergoing increased natural erosion, which has led to desertification of the entire region^14^.

Archaeological proxies (such as dates of abandonment layers and diets of animals) can be used to investigate the effects of any climatic or geomorphological shifts on patterns of human habitation in the Negev Desert. Bar-Oz *et al*.^25^ dated the collapse of the urban management of the Byzantine capital city of Elusa to the 6^th^ century CE, and correlated this event to the contemporary Late Antiquity Little Ice Age in Europe and the Justinian plague outbreak in 541 CE^26^. In their paper “Signs from a green desert”, Ramsay and Tepper analysed the contents of pigeon dung preserved in the dovecotes near Shivta to characterize the local environment around the settlement^27^. Due to the fact that dung was composed mainly of agricultural species (including fig, grape, olive, date, common pea and wild species that grow in fallow fields or on the edges of agricultural fields), they argue that the surrounding landscape was greener compared to today due to the presence of agricultural installations^27,28^.

Fried *et al*.^29^ found the micro-faunal assemblage of dovecote remains from the archaeological site of Sa’adon to be comparable to a modern micro-faunal assemblage from a desert region with higher levels of precipitation than modern-day Sa’adon. Based on this finding, they argue that a more productive agricultural system altered the surrounding environment to a degree that has not been seen until the 20^th^ century. The research presented herein aims to better characterize the nature of the vegetation in the vicinity of the settlements – was it concentrated in the agricultural spaces and thus restricted to the drainage channels, or was it more *expansive*, providing opportunities for animal grazing farther away from the settlements?

### Zooarchaeological and isotopic approach for understanding Negev climate during Antiquity

Three sets of stable isotopic proxies were measured from archaeological sheep and goat remains from Nessana and Shivta (Byzantine/Early Islamic) and Elusa (Byzantine):

1. Incremental tooth enamel carbonate δ^13^C and δ^18^O values (7–15 samples per tooth)
2. Sub-samples of tooth dentine collagen δ^13^C and δ^15^N values (2–4 samples per tooth)
3. Mandibular bone collagen δ^13^C and δ^15^N values (1 value per individual)

See Table 1 for a breakdown of the samples analyzed from each site and time period.

**Table 1:** Breakdown of samples analyzed in this study.

	Tooth enamel δ^13^C, δ^18^O values, and tooth dentine δ^13^C, δ^15^N values		Mandibular collagen δ^13^C, δ^15^N values	
Phase	Byzantine	Early Islamic	Byzantine	Early Islamic
*n =* (per site)	1 (Nessana)	5 (Nessana)	1 (Nessana)	5 (Nessana)
	9 (Elusa)		4 (Elusa)	
	3 (Shivta)			
Total	13	5	5	5

The combined proxies provide an opportunity to assess any changes in dietary and mobility behaviour of the animals that may have been caused by climatic fluctuations in this region. Incremental δ^18^O values provide insight into the geographical scope of the animals’ grazing behaviour. Variability in δ^18^O values of the ovicaprids is used to assess whether the animals’ grazing range was restricted to the immediate hinterlands of the settlements or whether they engaged in seasonal movement into higher altitudes or the Mediterranean coast in search of fresh graze. Since natural pasture would not have been available in the proximal landscape year round, if the animals’ movement was restricted, their diets would have to be supplemented by agricultural fodder. Fodder would likely be influenced by anthropogenic inputs (such as manure or other organic waste) used to increase the yields of agricultural crops^30,31^. These diets would thus serve as indicators of the soil conditions created by agricultural management. If, on the other hand, the animals engaged in long-range pastoralism – a scenario that is more representative of extreme desert environments, where it is more cost-effective to herd animals to locations of seasonal pasture than to cultivate fodder^32,33^ – it can be inferred that the animal diets were not influenced by anthropogenic factors. These diets would thus serve as a reliable proxy for understanding the climatic backdrop in the Byzantine/Early Islamic Negev Desert.

After establishing whether the animals provide dietary signatures of cultivated fodder or values of natural graze, carbon and nitrogen isotopic values are used to test hypotheses regarding the locations of ovicaprid pasture. Matching δ^13^C values from enamel and dentine provide information about the composition of their diet during the dry summers and the wet winters. The δ^15^N values help clarify whether the animals spent the entire year grazing within the arid Negev landscape, and if so, whether they grazed on diffused vegetation across the desert or contracted vegetation constrained to the *wadis*.

### Interpretative framework

Tooth enamel oxygen isotope values (δ^18^O) of domestic herbivores are precipitated in equilibrium with body water, which derives from ingested plant leaf dry matter, leaf water, and drinking water^34,35^. Due to fractionation effects, the δ^18^O values of body tissues are offset from the δ^18^O values of local precipitation, and species-specific equations are required to calculate the composition of the ingested meteoric water of measured animals. To date, no equations have been established for sheep or goats, so the results cannot be used to estimate the values of local precipitation or compared to the speleothem data. Instead, the patterns of seasonal changes in the δ^18^O composition of animal tooth enamel can be used to assess the animals’ grazing and mobility behavior.

In arid environments, enamel that mineralizes in the wet winter season (October–April) records the δ^18^O values of meteoric water and ingested plant leaf water, which vary by altitude due to the ‘rain-out effect’^36^. As a result, animals grazing in higher elevations will record lower δ^18^O values compared to animals grazing in valleys. In the dry summer season (May–September), when no rain falls, desert herbivores obtain water from plants and from water reservoirs such as cisterns and wells. Summer δ^18^O values of plant leaf matter are elevated due to the effects of increased temperature and evapotranspiration, which favor positive discrimination of ^18^O^37^. Water reservoirs built in bedrock shield the water from evaporation, so the δ^18^O values do not increase in the summer^38^.

Second and third molars of herbivorous animals mineralize along the axis of tooth growth, from the top of the crown to the enamel root junction. The process takes place over the first 12–18 months of the animals’ lives^39^. Sequential samples of δ^18^O values collected along the axis of tooth growth thus record the seasonal changes in δ^18^O inputs. Animals that ingest meteoric water and fresh plant matter during the winter and highly evapotranspired plant matter during the summer will record a sinusoidal annual sequence, with highest δ^18^O values corresponding to the dry season and lowest δ^18^O values reflecting the wet season^40–42^. Animals that ingest well-water during the summer or move to a location with fresh vegetation available in the dry season (such as the wetter Mediterranean coast) will record dampened intra-tooth variation in δ^18^O values. This is caused by the fact that δ^18^O values of well-water and plants from wetter regions are lower compared to δ^18^O values of desert dry season vegetation, which becomes enriched in ^18^O due to evapotranspiration^43^.

Plant carbon isotope values (δ^13^C) primarily reflect the mechanism in which carbon dioxide is fixed during photosynthesis. Plants employing the C_3_ photosynthetic pathway have δ^13^C values ranging from −34 to −21‰ (parts permil)^44^. C_4_ plants have δ^13^C values ranging from −17‰ to −9‰^44,45^. The δ^13^C values of C_3_ plants become more negative with increased water availability^46–48^, while the δ^13^C values of C_4_ plants can become less negative in water stress conditions, as in the case of two millet species^49^. Contracted vegetation growing in drainage channels tends to be dominated by the more water-demanding C_3_ plants, while diffuse vegetation shows mixed contribution of C_3_ and C_4_ plants^46^. As a result, the proportions of C_3_ vs. C_4_ vegetation in the diets of animals grazing in the desert can shed light on the type of vegetation cover – and thus the water availability – dominating the landscape in which the animals were grazing.

Le Houérou^50^ defines two types of vegetation cover in arid and desert regions. Diffuse vegetation can be found in Mediterranean and temperate climate steppes and consists of dwarf shrubs and/or perennial grasses, which are distributed regularly across the landscape. Contracted vegetation generally appears in regions with rainfall around 100 mm/yr and is made up of annual grasses that are confined to low-lying drainage channels receiving runoff water during the winter rain season.

Plants growing in the drainage channels have higher δ^15^N values compared to plants growing on exposed ridges due to increased production of gaseous forms of nitrogen during denitrification, which favors the release of the lighter ^14^N into the atmosphere^46^. Plants growing in the exposed ridges have low δ^15^N values due to the fact that they obtain nitrogen from the desert soil crust, which has been fixed by N_2_-fixing cyanobacteria^46,51^. The so-called ‘aridity effect’ used to explain higher δ^15^N values of plants from more arid regions (for e.g., Heaton^52^) is thus a reflection of biomass concentration in dry-washes during the wet season. Thus, animals grazing primarily in the drainage channels (which include fallow fields in agricultural terraces) have higher δ^15^N values than animals grazing on expansive (diffuse) vegetation. The degree of contracted vs. diffuse vegetation in the animals’ diets serves as an indicator of the water availability in the regions where the animals grazed.

The data do not provide the resolution to track the amounts of precipitation in the Levant during the time-period under investigation, but they can shed light on changes in vegetation cover in the Negev Desert towards the end of the Byzantine era through the Early Islamic period of this region (6^th^–7^th^ century CE). This provides the resolution to test whether the climatic trends in the wider region (e.g., decrease in precipitation recorded in the Soreq speleothem archive, Orland *et al*. 2009, and drop in the Dead Sea levels, Bookman *et al*. 2004) had an impact on the Negev micro-climate, and may have eventually led to the end of the Byzantine presence in this region in the 7^th^ century CE.

## Results

**Figure 2:**
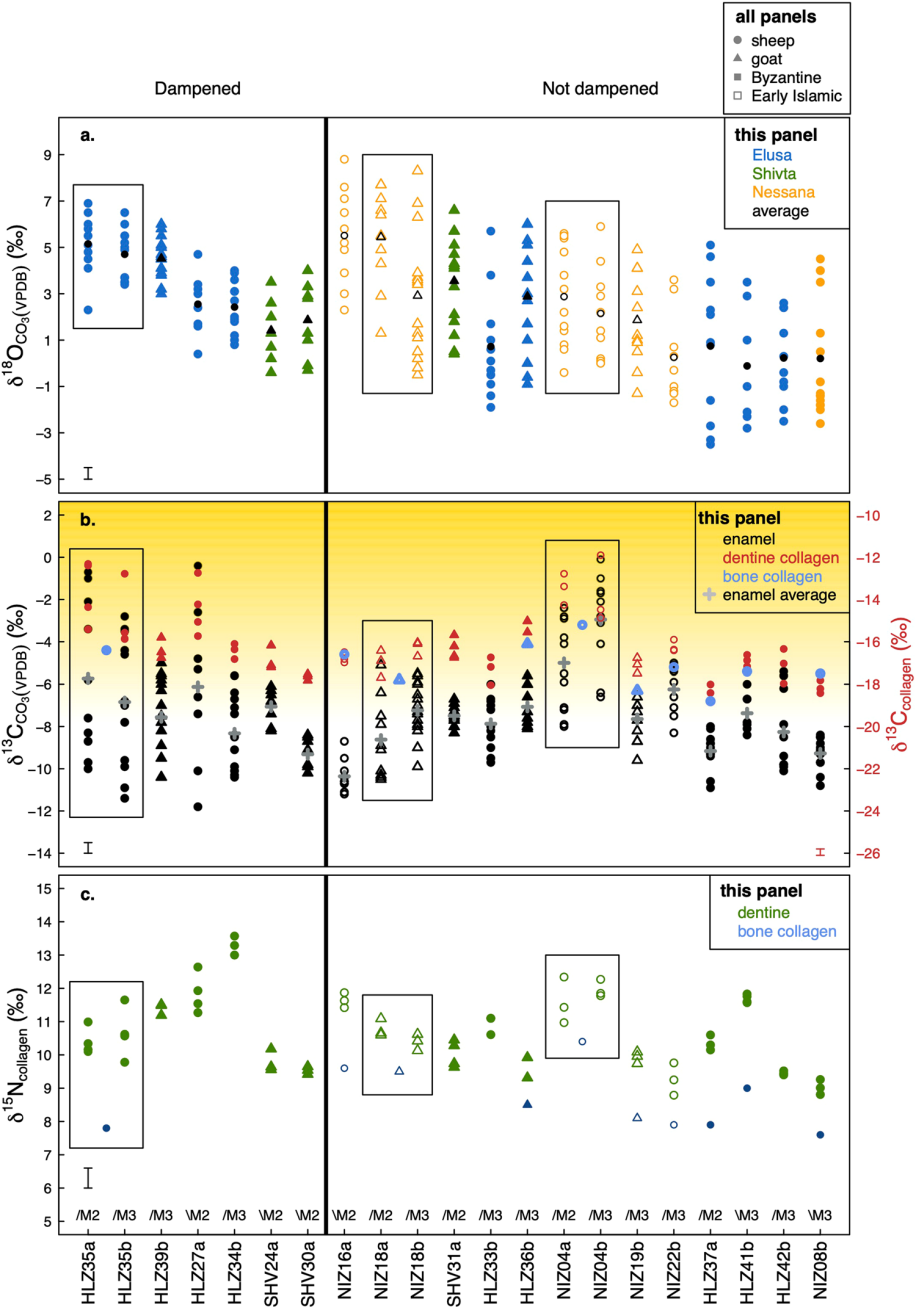
Results of stable isotopic measurements of all teeth carried out in this study. (**a**) δ^18^O values of tooth enamel carbonate. (**b**) δ^13^C values of tooth enamel carbonate, tooth dentine collagen and mandibular bone collagen. The shading indicates increasing contribution of C_4_ grasses in the herbivores’ diet, with the lower endpoint at −8‰ for carbonate δ^13^C values^44^ and −20‰ for collagen δ^13^C values. (**c**) δ^15^N values of tooth dentine collagen and mandibular bone collagen. Individuals were divided into groups based on the amplitude of δ^18^O variation (Δ^18^O). See text for details. M2 = second molar. M3 = third molar. / denotes lower molar. \ denotes upper molar. Boxes indicate the same individuals. Measurement error (pooled standard deviation of accuracy and precision, see Supplementary Files S1 and S2) shown in bottom left of panels A and C and bottom left and right of panel B.

All results are shown in Fig. 2. Raw data are presented in Supplementary Table S1 (enamel carbonate data), Table S2 (dentine collagen data) and Table S3 (mandibular collagen data). All figures in this paper were prepared using R (R Core Team, version 3.6.0). The sheep have been identified to species using Zooarchaeology by Mass Spectrometry (ZooMS).

Powdered enamel samples were extracted along the axis of tooth growth (1 mm wide samples taken 1 mm apart). Total number of sub-samples taken per tooth ranged between 7 and 15, depending on the crown height. The δ^18^O values of all samples range from −4.6‰ to +8.8‰ (see Fig. 2a). The amplitudes of intra-individual variation in δ^18^O values (denoted with Δ^18^O) range from +3.0‰ to +8.8‰ (mean +5.6‰). See Supplementary Table S4 for summary statistics of the enamel carbonate data. Most individuals exhibit sinusoidal sequences of annual variation in δ^18^O values (see Supplementary Figs. S2–S5 for all data graphed with respect to tooth position). These individuals have Δ^18^O values above 4.5‰. Six individuals (all from the Byzantine period) exhibit dampened amplitudes of variation below 4.5‰. There is a statistically significant difference at 99% confidence between the δ^18^O amplitudes of the dampened and non-dampened group (unpaired unequal variance student’s t-test, *t* = −7.240, *p* < 0.01; mean amplitude of dampened group = 3.8‰, mean amplitude of non-dampened group = 6.7‰). Three individuals had both second (M2) and third (M3) molars measured and the Fig. 2 shows that there is a close correlation between the matching pairs of sequences.

Animals from Nessana record slightly lower δ^18^O values compared to the ovicaprids from the other sites, but this difference is not statistically significant (Shapiro test showed that the data is not normally distributed, W = 0.982, p < 0.005; Kruskal-Wallis test showed that there is no significant difference between the mean δ^18^O values of the three groups, H(2) = 3.049, *p* = 0.218). There is a statistically significant difference between the δ^18^O values of sheep and goats (mean sheep δ^18^O = 2.1 ± 2.7‰, n = 137; mean goat δ^18^O = 3.2 ± 2.3‰, n = 93; unpaired unequal variance student’s t-test, *t =* −3.24, *p* < 0.01), which is likely caused by their distinct dietary adaptations. Sheep are grazers and require grasses to obtain their required nutrition, while goats are browsers and can survive on more woody, dry and high-fibrous diet^53^. There is no systematic difference between the sheep and the goats in terms of their amplitudes of δ^18^O variation. Three sheep and three goats have dampened annual sequences, while 7 sheep and 5 goats do not.

**Figure 3:**
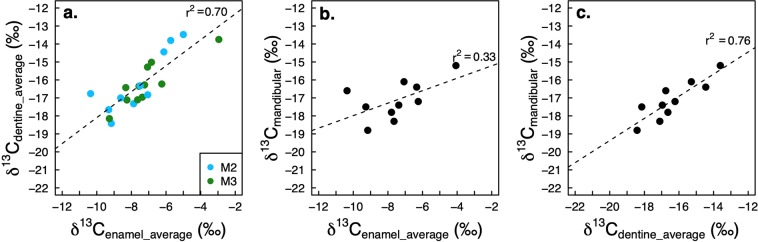
Linear correlations between the three δ^13^C proxies from each individual. (**a**) average δ^13^C_enamel_ vs. average δ^13^C_dentine_, (**b**) average δ^13^C_enamel_ vs. 𝛿^13^C_mandibular bone collagen_, (**c**) average δ^13^C_dentine_ vs. δ^13^C_mandibular bone collagen_.

δ^13^C values of animals from this study were obtained from both the organic and the inorganic fractions of teeth, and from the mandibular bone (from individuals where the bone was preserved). Supplementary Table S5 presents the summary statistics for the dentine samples from each tooth. Figure 3 shows the correlation between the three sets of δ^13^C values obtained from each tooth. Figure 3a shows that the δ^13^C_dentine_ and δ^13^C_enamel_ values are positively correlated (r^2^ = 0.70). Bulk bone collagen δ^13^C values provide a dietary signature that post-dates the tooth formation period. Figure 3b,c shows that the mandibular bone collagen δ^13^C values are better correlated with the dentine δ^13^C values (r^2^ = 0.76) than they are with enamel δ^13^C values (r^2^ = 0.33). This is consistent with a slightly later formation period of dentine during tooth development^39^.

Figure 2b shows the δ^13^C values of all teeth measured in this study. The shading indicates the degree of contribution of C_4_ vegetation in the animals’ diets, with the lower endpoint of C_4_ input at −8‰ for carbonate values and −20‰ for collagen values (cf Cerling^44^). All animals consumed a mixed diet of C_3_ and C_4_ plants, with three individuals (HLZ27, HLZ35 and NIZ04) subsisting on a high amount of C_4_ vegetation for part of tooth formation period. Eleven individuals have mixed C_3_−C_4_ average enamel carbonate δ^13^C values (located above the −8‰ line) and ten individuals have purely C_3_ average enamel carbonate δ^13^C values. No individual dentine δ^13^C measurement is situated below the C_3_-C_4_ endpoint line, while all individuals have enamel δ^13^C values below this line. This is likely the result of the sampling resolution (the enamel sequences have 7 to 15 sub-samples, while the dentine sequences have 2 to 4 samples per tooth). Three individuals (NIZ16, SHV31 and NIZ08), none of which engaged in cross-altitudinal mobility, may have been foddered. They exhibit flat δ^13^C sequences, which do not track seasonal response to water and temperature fluctuations of fresh vegetation (cf Makarewicz^54^) (see Supplementary Figs. S3 and S5).

**Figure 4:**
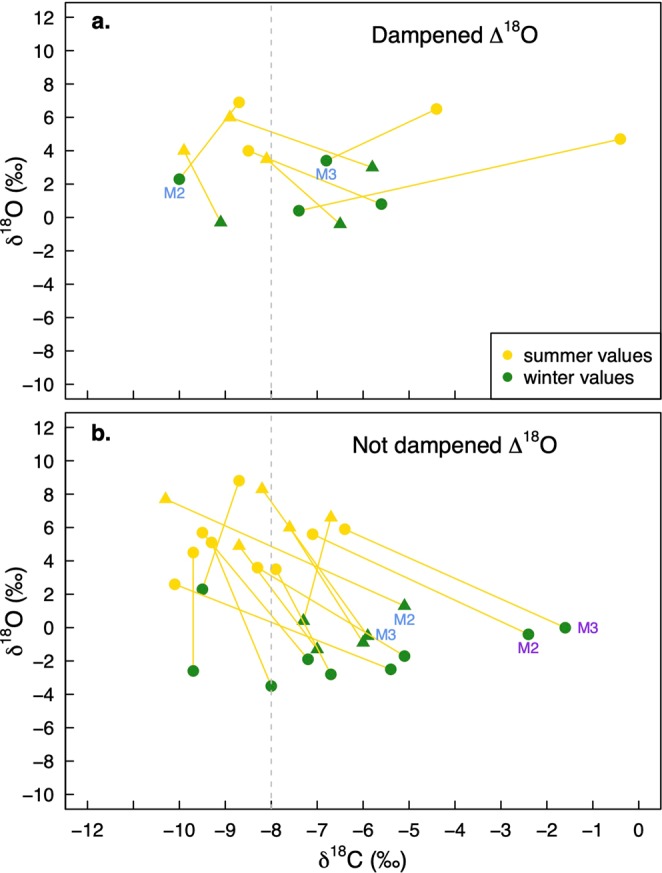
Matching summer and winter δ^13^C and δ^18^O values of all individuals. Seasonality was determined using δ^18^O_max_ values for summer and δ^18^O_min_ values for winter for each individual. M2 and M3 labels of matching colours denote the same individuals. Individuals were divided into groups based on the amplitude of intra-tooth δ^18^O variation (Δ^18^O). (**a**) Dampened Δ^18^O (below 4.5‰), (**b**) Not dampened Δ^18^O (above 4.5‰).

There is no statistically significant difference between enamel carbonate δ^13^C values of the sheep and the goats (mean sheep = −7.3 ± 2.8‰, mean goat = −7.7 ± 1.4‰; unpaired unequal variance student’s t-test, *t* = 1.37, *p* = 0.171). There is a small yet statistically significant difference (99% confidence) between all dentine δ^13^C values of all sheep and goats (mean sheep = −15.9 ± 1.8‰, mean goat = −16.6 ± 0.7‰; unpaired unequal variance student’s t-test, *t* = 3.23, *p* < 0.01), but no difference between their mandibular collagen δ^13^C values (mean sheep = −17.0 ± 1.1‰, mean goat = −17.4 ± 1.2‰; unpaired equal variance student’s t-test, *t* = 0.50, *p* = 0.632). Figure 4 shows the summer and winter enamel carbonate δ^13^C values. The animals are divided into two groups according to their amplitude of intra-tooth δ^18^O variation (discussed above). There is no systematic difference between the summer and winter diets of the sheep and the goats.

Figure 2c shows the sequential δ^15^N values from tooth dentine and bulk δ^15^N values of mandibular bone collagen of animals measured in this study. The raw data are presented in Supplementary Tables S2 and S3. The δ^15^N values of all samples range from +7.5 to +14‰. There is a minute but statistically significant difference between the dentine δ^15^N values of sheep and goats (mean sheep = 11.0 ± 1.2‰, mean goat = 10.2 ± 0.6‰; unpaired unequal variance student’s t-test, *t* = 4.73, *p* < 0.01), but no difference between their mandibular collagen values (mean sheep = 8.6 ± 1.1‰, mean goat = 8.7 ± 0.7‰; unpaired unequal variance students’ t-test, *t* = −0.17, *p* = 0.870).

### Lack of chronological shift

**Figure 5:**
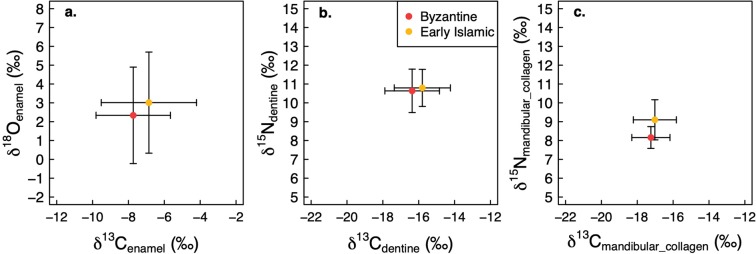
Stable isotopic measurements from each archaeological proxy (enamel carbonate, tooth dentine and mandibular collagen) divided into chronological phases. (**a**) δ^13^C_enamel_ vs. δ^18^O_enamel_, (**b**) δ^13^C_dentine_ vs. δ^15^N_dentine_, (**c**) δ^13^C_mandibular collagen_ vs. δ^15^N_mandibular collagen_.

The data presented in this study suggest that there were no significant chronological shifts in the diets of domestic sheep and goats between the Byzantine and the Early Islamic periods at Nessana, Elusa and Shivta. All lines of evidence – tooth enamel δ^13^C/δ^18^O values, tooth dentine δ^13^C/δ^15^N values and mandibular bone collagen δ^13^C/δ^15^N values – show statistically similar values for the two phases (Fig. 5, see Table 2 for results of individual t-tests; t-tests were carried out instead of multi-variate analyses because the data come from three separate datasets).

**Table 2:** Results of statistical analyses comparing the three sets of stable isotopic proxies from each chronological phase.

	variance	*t*=	*p*=	Byzantine			Early Islamic		
				no. of individ.	no. of sub-samples	mean ± 1σ (in ‰)	no. of individ.	no. of sub-samples	mean ± 1σ (in ‰)
**Enamel carbonate**									
δ^13^C	unequal (7.7 vs. 1.9)	−2.52	0.013	13	153	−7.7 ± 2.1	5	77	−6.9 ± 2.7
δ^18^O	unequal (7.4 vs. 5.3)	−1.83	0.070	13	153	2.3 ± 2.6	5	77	3.0 ± 2.7
**Dentine collagen**									
δ^13^C	unequal (3.3 vs. 0.5)	−1.96	0.054	13	45	−16.4 ± 1.5	5	21	−15.8 ± 1.6
δ^15^N	unequal (1.5 vs. 0.4)	−0.81	0.421	13	45	10.6 ± 1.2	5	21	10.8 ± 1.0
**Mandibular bone collagen**									
δ^13^C	equal (1.2 vs. 1.3)	−0.31	0.767	5	5	−17.2 ± 1.1	5	5	−17.0 ± 1.2
δ^15^N	unequal (1.2 vs. 0.5)	−1.73	0.132	5	5	8.2 ± 0.6	5	5	9.1 ± 1.1

Individual t-tests were carried out on each pair of δ^13^C, δ^18^O and δ^15^N values because the data came from three different datasets (enamel carbonate, dentine collagen and mandibular bone collagen), which could not be combined to carry out multi-variate analyses.

## Discussion

### δ^18^O values indicate that the animals grazed in a wide landscape

The measured ovicaprid δ^18^O values are higher compared to those of archaeological gazelles from Amud cave^55,56^. This is the result of the fact that the animals in this study lived in a drier microclimate, where vegetation was more prone to evapotranspiration favoring positive fractionation^36^. The large variability in wet season signatures suggests that the animals grazed in a wide landscape not limited to the immediate surroundings of the settlements.

Individuals that exhibit non-dampened seasonal signals may have spent the entire year at variable altitudes in the Negev desert, recording evapotranspired plant δ^18^O values in the summer. The animals with the lowest δ^18^O_min_ values (reaching down to −3/−4‰) likely grazed at the highest elevations during the winter compared to all the other measured animals. Those with the highest winter δ^18^O_min_ values (reaching up to +8/+9‰) likely grazed at low altitudes either around the modern-day city of Be’er Sheva or in the Arava Valley below sea level. Individuals with dampened amplitudes may have consumed significant amounts of well-water during the summer or migrated to the Mediterranean zones in search of fresh vegetation during the dry season.

Overall, the δ^18^O results indicate that the ovicaprids from Shivta, Nessana and Elusa did not all graze in the immediate hinterlands of the sites all year round. Some of the animals may have moved to the wetter Mediterranean zone in search of fresh vegetation during the summer dry season. Alternatively, they may have stayed in the desert and ingested ample amounts of water from closed cisterns, which would have dampened their seasonal δ^18^O signal. The rest of the animals spent both seasons at either low, mid or high altitudes in the arid region. The results of the δ^13^C and δ^15^N analyses will help constrain the extent of seasonal mobility that the animals partook in. The data will aid in defining likelihood that the animals were taken outside of the Negev Desert during the summer, as they have been by nomadic Bedouin in the 20^th^ century^57^.

### δ^13^C values indicate mixed C_3_−C_4_ diets with summer diets dominated by C_3_ grasses

Sequential tooth enamel carbonate δ^13^C values match the δ^18^O sequences discussed above and provide an opportunity to determine the composition of vegetation consumed during the summer dry season (when the δ^18^O values are at their maximum) and the winter wet season (when the δ^18^O values are at their minimum). The results show that the diets of the animals that do not have dampened Δ^18^O values (i.e., that did not consume significant amounts of well-water or migrate to the Mediterranean coast in the summer) had mixed C_3_−C_4_ diets in the winter and predominantly C_3_ diets in the summer. Three out of five individuals with dampened Δ^18^O values follow the same trend, while the other two record higher inputs of C_4_ vegetation in the summer. This supports the inference that the dampened amplitude of intra-tooth variation in δ^18^O values was caused by drinking of water from reservoirs, which would be necessary to supplement a salty diet of C_4_ desert chenopods. As individuals with the least dampened signals (i.e., largest Δ^18^O signals) also have the lowest δ^18^O_min_ values, the data indicates that water-supplementation was carried out in lower topographies, where winter δ^18^O values of precipitation are lower^36^. Individuals that consumed predominantly C_3_ diets in the winter also consumed C_3_ vegetation in the summer, with no animal recording a shift to C_4_ vegetation in the summer.

Overall, the δ^13^C data suggest that most of the animals consumed C_3_ short-lived annuals (*Therophytes*) available in the desert *wadis* in the summer. A small number of individuals consumed a larger amount of C_4_ plants in the dry season and supplemented this with drinking water from closed cisterns. Some animals may have either also consumed reservoir water or migrated to the Mediterranean zone in the summer, while consuming a diet dominated by C_3_ vegetation. The δ^15^N data presented in the next section will help constrain the degree of movement within or outside of the desert region.

### δ^15^N values show that the ovicaprids grazed in desert wadis all year round

The measured δ^15^N values are most comparable to δ^15^N values of modern desert gazelles and ibex (reaching +16‰) in eastern Mediterranean regions with high aridity index, where rainfall is low and evapotranspiration is high — similar to the climate in the Negev Desert^58^. Modern plant samples collected at rainfall zones below 100 mm/yr in modern-day Israel record values between −1‰ and +9‰, with those growing in exposed ridges falling between −1‰ and +3‰ and those growing in drainage channels ranging from +3‰ to +9‰ (Hartman and Danin^46^, their Fig. 2). δ^15^N values decrease in regions with higher mean annual rainfall^46,59,60^. When the average diet–tissue_collagen_ fractionation offset of 3–5‰^61,62^ is subtracted from the collagen values obtained in this study, the estimated δ^15^N value of the plants consumed by the ovicaprids is +4 to +10‰. This is consistent with the animals exclusively eating vegetation growing in the *wadis*. Based on these results, it is argued herein that the Byzantine and Early Islamic ovicaprids excavated at Nessana, Elusa and Shivta did not eat expansive (diffused, cf Le Houérou^50^) vegetation, but vegetation restricted to the seasonally flooded drainage channels.

### Persistance in grazing behavior and management strategies points to stability in the local Negev microclimate

The dietary and mobility patterns of sheep and goats from Elusa, Nessana and Shivta provide information about the climatic opportunities that constrained the herding management in the Negev Desert during the Byzantine and Early Islamic periods. Sequential tooth enamel carbonate δ^18^O values show that the ovicaprids did not obtain their summer and winter graze within the hinterlands of the towns but grazed within the wider landscape over the course of the changing seasons. This finding is consistent with the results of archaeobotanical analysis of animal dung from Early Islamic Shivta, which indicated that the livestock moved out of the Negev Highlands to “sample other phytogeographic zones” such as the Western and Northern Negev^63^. Because most of the animals engaged in seasonal mobility, it is inferred that their pasture was composed of naturally growing vegetation rather than agricultural products, and their diets thus serve as a proxy for understanding the composition of desert vegetation coverage during the time period in question.

In their survey of Bedouin plant use in the Sinai and the Negev, Bailey and Danin^32^ observed that in the dry summer season, the animals of this region consume dwarf shrubs (chamaephytes) such as *Artemisia herba-alba*, *Artemisia monosperma*, *Gymnocarpos decander*, and *Noaea mucronate* (C_4_), perennial plants including *Centaurea aegyptiaca* and *Foeniculum vulgare*, summer annuals such as *Salsola inermis* (C_4_) and lichen (*Ramalina maciformis*). After the annuals and the perennials have dried up, they turn to less preferred choices of dwarf shrubs and shrubs (phaneorphytes), including *Achillea fragrantissima*, *Atriplex halimus* (C_4_), *Hammade negevensis* (C_4_), *Hammade scoparia* (C_4_), *Zygophyllum dumosum*. Consumption of small quantities of salty and oxalic acid-rich C_4_ plants (chenopods) – which needs to be supplemented by steady supplies of drinking water – helps the animals eliminate intestinal parasites that they caught through consumption of annuals earlier in the year.

Sequential tooth enamel carbonate and tooth dentine δ^13^C values show that sheep and goats analyzed in this study consumed a mixed diet of C_3_ and C_4_ plants. Two sheep and one goat may have been foddered for part of the year, but the remaining animals subsisted on fresh vegetation in the areas where they grazed. The summer diets were dominated by the more water-demanding C_3_ vegetation but high δ^15^N values indicate that the animals spent the entire year within the arid Negev Desert, and thus did not migrate towards the wetter Mediterranean zones, as they did in more recent years^57^. As summer C_3_ plants are restricted to ‘contracted’ vegetation growing in the low-lying *wadi* channels (cf Le Houérou^50^), the animals’ grazing patterns were thus constrained to drainage areas of the desert region. This is consistent with the archaeobotanical findings from Shivta, which included reed and sedge phytoliths (i.e., plants that grow close to water sources such as cisterns, springs and pools) in the dung of Early Islamic livestock at this site^63^.

Herding of the animals across the desert landscape was likely managed by nomadic pastoralists, who are known to travel varying distances to bring their animals to available graze on a seasonal mobility schedule. The Bedouin in the Sinai highlands and in the Galilee region travel a few kilometers every year^64,65^. Other communities, such as the Al-Murrah in the Arabian Peninsula and the Basseri moving between the Zagros mountains and the Persian Gulf lowlands, can traverse 400–800 km in a season^66^. The reasons for this movement are several, including availability of pasture, market demands, tribal boundaries, state laws, tribal factors such as wealth, status and size of the community, and negotiations with landowners^33,67^.

In the early part of the 20^th^ century, the Bedouin who have been herding their animals in the Negev Desert established a seasonal cycle that took them from the Dimona valley in the northern Negev during the wet season to as far as the Jezreel Valley c.200 km north during severe drought years^57^. During the Byzantine period, local semi-nomadic or nomadic groups, likely Arab-Semitic in nature – and possibly including the known Saracens groups – were among the population who inhabited Byzantium’s eastern provinces and were thought to have partaken in desert pastoralism^68,69^. The fact that the animals were not taken outside of the arid region suggests that the farmers in the Mediterranean zones did not develop an interdependent relationship with the desert pastoralists, where the animals would be allowed to graze on fallow fields and fertilize the fields for the upcoming sowing season – a relationship that has been documented around the Mediterranean world in traditional farming settings^70^. Instead, the interactions are likely to have been delimited by land ownership policies that did not allow the nomadic pastoralists from the desert to exploit pasture grasses north of the desert during the dry season.

The results of this study show that between the 6^th^–7^th^ centuries CE, precipitation in the Negev was not high enough to turn the desert into a “green desert” covered by *diffused* vegetation (cf Le Houérou^50^). The water flow in the drainage channels was higher than it is today^14^, but the plant biomass production was constrained to these low-lying topographies. The *wadis* are where the pigeons from Shivta obtained their agricultural feed (cf Marom *et al*.^71^, Ramsay *et al*.^28^, Ramsay and Tepper^27^), where higher populations of jirds (*Meriones*) identified in the micro-faunal assemblage from Sa’adon developed (cf Fried *et al*.^29^, Tepper *et al*.^72^). This is where sheep and goats from Elusa, Nessana and Shivta obtained their C_3_ vegetation during the dry summer months. These findings indicate that even though localized environmental deteriorations were causing societal decline elsewhere at the same time (for e.g. the Late Antiquity Little Ice Age in Europe^73^), the Northern Negev Desert did not experience an abrupt downturn that would have affected the dietary behavior of animals that spent the entire year in the desert.

The lack of significant climate deterioration in the 6^th^–7^th^ century CE points to other drivers responsible for the abandonment of the Byzantine Negev settlements. One such catalyst is the collapse of the trade patterns that connected the Arabian Peninsula with the Mediterranean world, and of which the Negev Desert was a significant route. Another possible reason was a decline in Byzantine population caused by the Justinian plague, which would have altered the demand for production of exotic goods within the empire. From the animals that were investigated in this study, only Byzantine individuals showed evidence of consumption of water from wells and/or closed reservoirs. Although the sample size for this observation is very limited, it is possible that these water features came out of use in the Early Islamic period, leading to abandonment of the agricultural installations. The findings presented herein indicate that the end of the Byzantine presence in the Negev Desert was not as dramatic as previously suggested, with a catastrophic climatic shift leading to a societal collapse. Instead, the settlement abandonment was more likely the result of reorganization of economic and/or territorial priorities within the wide Byzantine empire.

## Materials and Methods

Sheep and goat samples were obtained from archaeological assemblages in the recently re-excavated sites of Subeita (Shivta), Nessana (Nitzana) and Elusa (Halutza). Chronological assignments (Byzantine vs. Islamic period) were made based on ceramic and coin typologies and confirmed using associations to radiocarbon-dated contexts (Elusa: Bar-Oz *et al*.^25^; Shivta: Tepper *et al*.^12^; Nessana: forthcoming). Material from transitional periods was not included. Prior to isotopic analysis, all teeth were identified to species using Zooarchaeology by Mass Spectrometry (ZooMS) whereby 1–2 mg collagen was digested with trypsin for ~18 h in 50 mM ammonium bicarbonate, ziptipped, and analysed by an Ultraflex II Matrix Assisted Laser Desorption Ionization Time of Flight mass spectrometer following dried droplet crystalisation with 10 mg/mL alpha-cyano hydroxycinnamic acid in 50% acetonitrile/0.1% trifluoroacetic acid^74^. Confirmation of species was established via published caprine peptide biomarkers^74,75^.

### Tooth enamel carbonate

Tooth enamel carbonate δ^13^C and δ^18^O values were obtained from 32 teeth (26 individuals). During instrumental analysis, a number of the measurements did not provide reliable results. Reliable values were obtained for 22 full sequences (20 individuals) and 10 partial sequences (10 individuals). Because the partial sequences do not record complete annual cycles, their maximum and minimum values do not represent the ‘real’ maximum and minimum values for the respective teeth. Similarly, their average values present skewed results (biased towards the portion of the tooth for which the results exist). For this reason, only the full sequences are included in the analysis.

Prior to sampling, the external surface of the teeth was cleaned using a laboratory Dremel tool with a diamond drill bit. 5–10 mg sub-samples of powdered enamel were collected at 1 mm intervals along the axis of growth from the buccal side of each tooth, starting at the occlusal surface and ending at the enamel root junction (erj), following the protocol established by Balasse^76^ (see Supplementay Fig. S1 for an image of a sampled set of M2 and M3 teeth).

Powdered enamel samples were pre-treated with 2–3% NaOCl for 24 hours, washed 3 times in distilled water and subsequently soaked in 0.1 M acetic acid for 4 hours before being rinsed 3 times in distilled water (following Balasse *et al*.^77^). Measurements were carried out at the Geological Survey of Israel. Powdered samples were placed at the bottom of clean vacuum vessels and a few drops of dry (100%) phosphoric acid (H_3_PO_4_) were added at the top. The vessels were then positioned horizontally and flushed with pure helium for 10 minutes in order to remove atmospheric CO_2_. After flushing, the sample and acid were allowed to interact, releasing CO_2_ into the sealed vessels.

Stable oxygen and carbon isotopic compositions were measured using a Finnigan Gas Bench II extraction system attached to a ThermoFinnigan Delta PLUS XP continuous flow mass spectrometer at the Department of Geochemistry at the Geological Survey of Israel, Jerusalem. δ^13^C and δ^18^O values were calibrated relative to VPDB scales using an internal Carrara Marble standard (δ^13^C = +4.74‰, δ^18^O = −3.85‰), which had been calibrated over the long-term against international standards NBS-19 (δ^13^C = +1.95‰, δ^18^O = −2.20‰) and NBS-18 (δ^13^C = −5.01 ± 0.34, δ^18^O = −23.2 ± 0.1‰). Measurement uncertainty was also monitored using the Carrara Marble standard, and calculated following the standardized procedure outlined in Szpak *et al*.^78^. Precision (*u(Rw)*) was determined to be ±0.43‰ for δ^13^C and ±0.46‰ for δ^18^O on the basis of repeated measurements of the Carrara Marble standard placed in the analytical runs every 8 samples and on the measurements of sample replicates. Accuracy (systematic error, *u(bias)*) was determined to be ±0.16‰ for both δ^13^C and δ^18^O on the basis of the difference between the observed and known 𝛿 values of the internal standard. The systematic error in these analytical runs (tooth enamel samples with c.2–5% carbonate content) was higher compared to the systematic error in analytical runs of samples with higher (i.e. >90%) carbonate content at this laboratory (average long-term accuracy ±0.1‰). Using the equations in Appendix F from Szpak *et al*.^78^, the total analytical uncertainty was estimated to be ±0.46‰ for δ^13^C and ±0.49‰ for δ^18^O. Additional details are provided in Supplementary Data File S1.

### Tooth dentine and mandibular collagen

Tooth dentine collagen δ^13^C and δ^15^N values were obtained from 31 teeth (25 individuals). Two teeth (from one individual) only yielded a single pair of δ^13^C and δ^15^N measurements. The rest of the teeth yielded between 2–4 measurements per tooth. The lobes of teeth that were previously sampled for tooth enamel were sectioned into 5–10 mm chunks. The chunks were then demineralized in 0.5 M HCl and subsequently hydrolyzed for collagen extraction following the modified Longin method^79^. Bone collagen was extracted from 19 mandibles (the rest of the teeth analyzed sequentially were found loose in the archaeological matrix) using the same method.

Stable carbon and nitrogen isotopic compositions were determined using an IsoPrime GVI Isotope Ratio Mass Spectrometer coupled to a Euro-Vector Elemental Analyzer at the Department of Biology at Boston University. δ^13^C values were calibrated relative to VPDB using internal standards glycine (δ^13^C = +33.98 ± 0.10) and peptone (δ^13^C = −14.75 ± 0.09), which had been calibrated over the long-term against NBS-20 (δ^13^C = −1.06‰), NBS-21 (δ^13^C = −28.16‰), and NBS-22 (δ^13^C = −30.03‰). δ^15^N values were calibrated relative to AIR using the same internal standards, calibrated over the long-term against IAEA-N1 (δ^15^N = +0.4 ± 0.20‰), IAEA-N2 (δ^15^N = +20.3 ± 0.20‰) and IAEA-NO3 (δ^15^N = +4.7 ± 0.2‰). Precision (*u(R(w)*) was determined to be 0.23‰ for δ^13^C and 0.47‰ for δ^15^N on the basis of repeated measurements of the check standards. Accuracy (systematic error, (*u(bias)*) was calculated using the same long-term averages to be 0.10‰ for δ^13^C and 0.38‰ for δ^15^N on the basis of the difference between the observed and known 𝛿 values of the check standards and the long-term standard deviations of these standards. Using the equations presented in Appendix F^78^, the total analytical uncertainty was estimated to be ±0.27‰ for 𝛿^13^C values and ±0.61‰ for δ^15^N values. See Supplementary Data File S2 for additional details.

## Supplementary information

Supplementary information.

Supplementary information 2.

Supplementary information 3.

Supplementary information 4.

Supplementary information 5.

Supplementary information 6.

## Data Availability

All data gathered in this study are presented in this paper and its Supplementary Materials.
