# Supplementary material for: Climate stability and societal decline on the margins of the Byzantine empire in the Negev Desert
Source: Sci Rep. 2020 Jan 30;10:1512. doi: 10.1038/s41598-020-58360-5 (PMC6992700; doi:10.1038/s41598-020-58360-5)
Supplement: Supplementary file 1 — Supplementary information. [file 41598_2020_58360_MOESM1_ESM.pdf]

Climate stability and societal decline on the margins of the Byzantine empire in the Negev desert

Authors
Petra Vaiglova, Gideon Hartman, Nimrod Marom, Avner Ayalon, Miryam Bar-Matthews, Tami Zilberman, Gal Yasur, Michael Buckley, Rachel Bernstein, Yotam Tepper, Lior Weissbrod, Tali Erickson-Gini, Guy Bar-Oz

Supplementary Fig. S1 Photograph of a sampled tooth. Sampling was carried out on the buccal side of herbivore second and third molars. In this example, the M3 was sampled on two cusps for comparative purposes. Photograph by Mason Seymore.

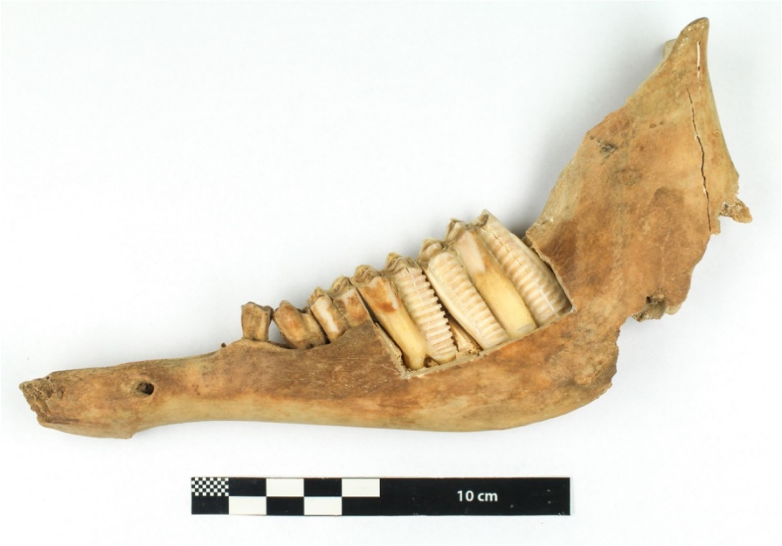

Supplementary Fig. S2. Matching stable isotopic sequences of individual teeth carried out in this study. Color coded y-axes indicate the scales for δ^{13}C of dentine collagen (red), δ^{13}C of tooth enamel carbonate (black), δ^{18}O of tooth enamel carbonate (blue) and δ^{15}N of tooth dentine collagen (green).

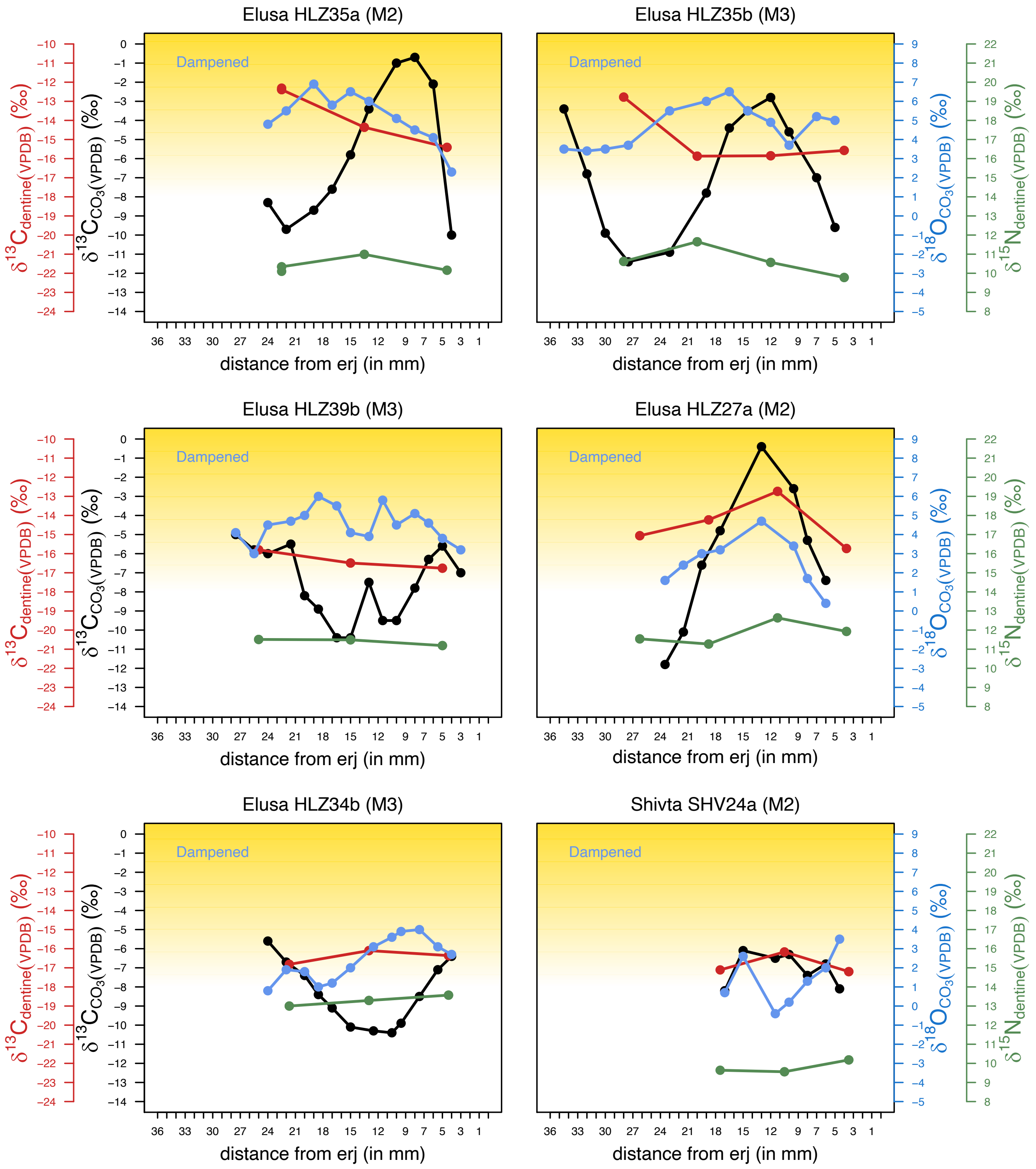

Supplementary Fig. S3. Matching stable isotopic sequences of individual teeth carried out in this study. Color coded y-axes indicate the scales for δ^{13}C of dentine collagen (red), δ^{13}C of tooth enamel carbonate (black), δ^{18}O of tooth enamel carbonate (blue) and δ^{15}N of tooth dentine collagen (green).

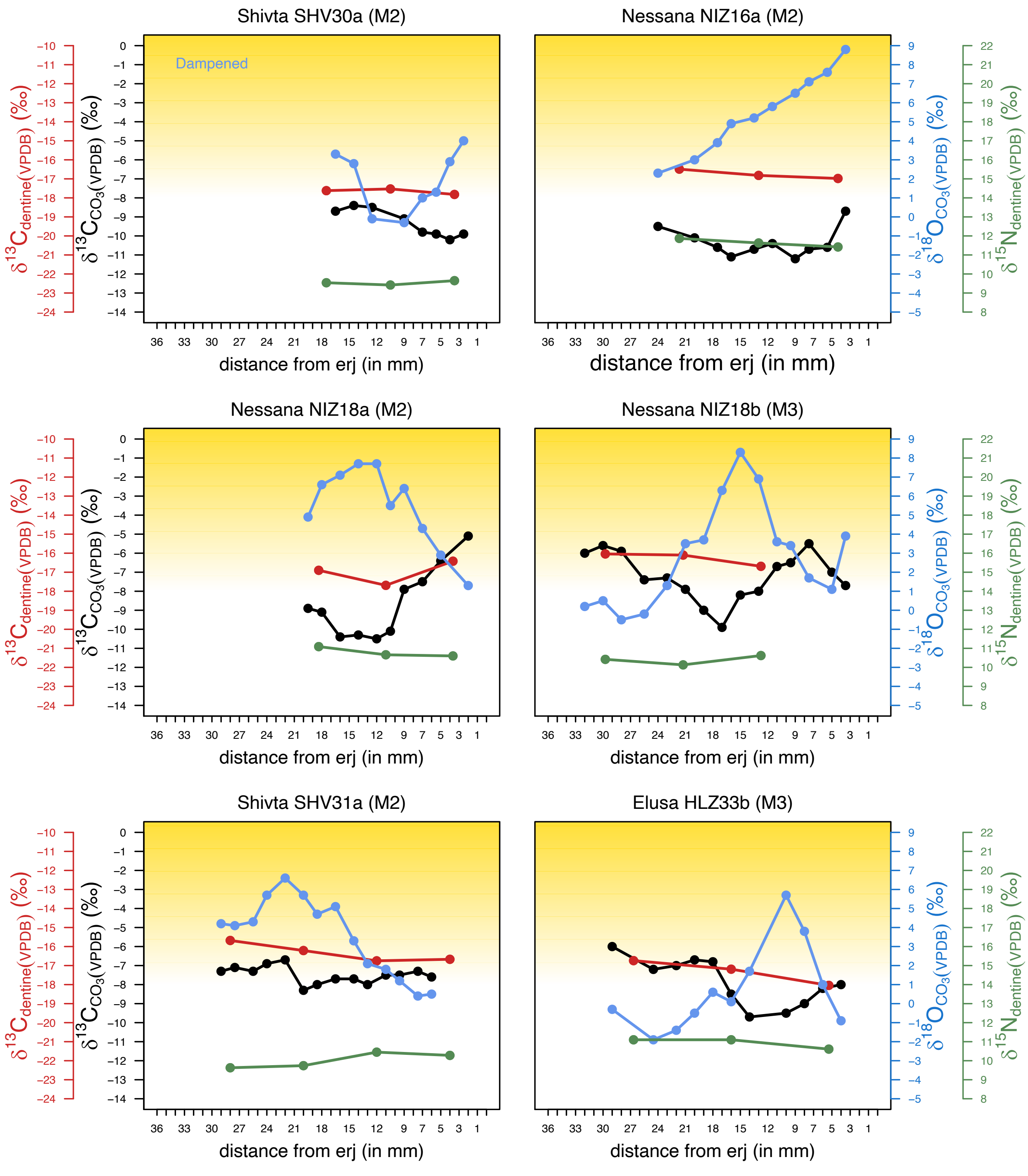

Supplementary Fig. S4. Matching stable isotopic sequences of individual teeth carried out in this study. Color coded y-axes indicate the scales for $\delta^{13}C$ of dentine collagen (red), $\delta^{13}C$ of tooth enamel carbonate (black), $\delta^{18}O$ of tooth enamel carbonate (blue) and $\delta^{15}N$ of tooth dentine collagen (green).

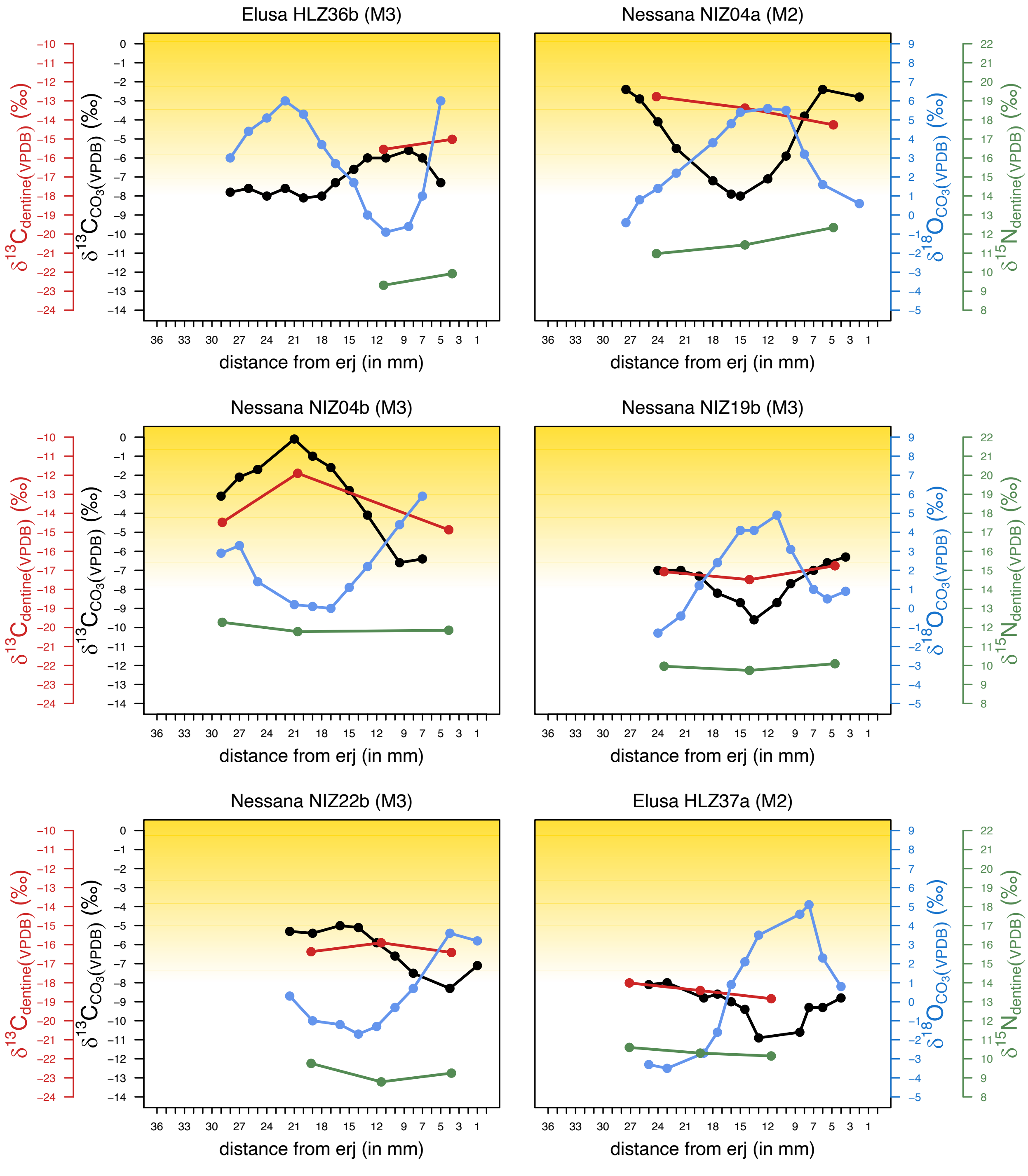

Supplementary Fig. S5. Matching stable isotopic sequences of individual teeth carried out in this study. Color coded y-axes indicate the scales for δ^{13}C of dentine collagen (red), δ^{13}C of tooth enamel carbonate (black), δ^{18}O of tooth enamel carbonate (blue) and δ^{15}N of tooth dentine collagen (green).

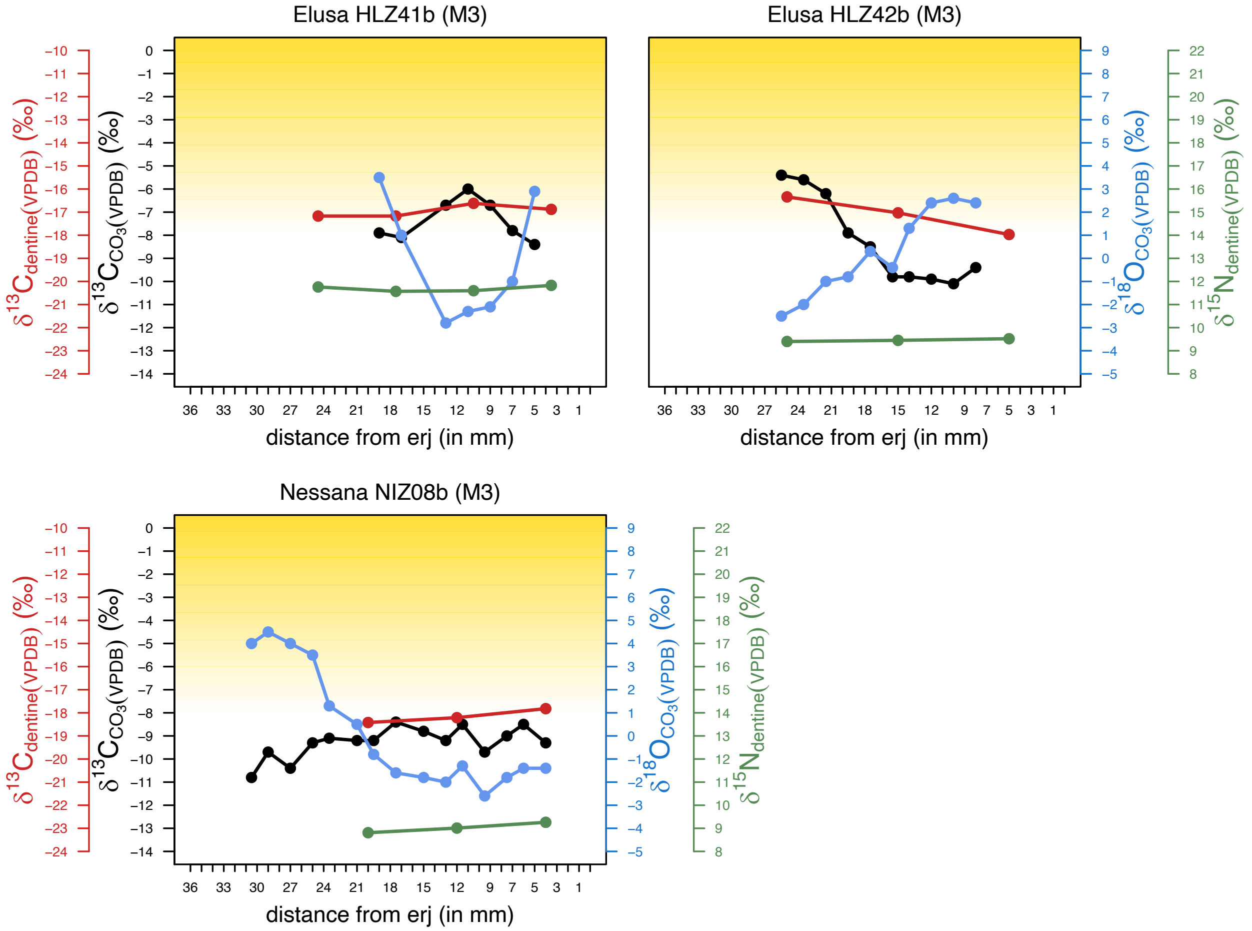

Supplementary Data File S1 – Calibration and Analytical Uncertainty for Enamel Isotopic Measurements ($\delta^{13}C$ and $\delta^{18}O$)

Carbon and oxygen isotopic compositions were determined using Finnigan Gas Bench II extraction system attached to a ThermoFinnigan Delta PLUS XP continuous flow mass spectrometer at the Department of Geochemistry at the Geological Survey of Israel, Jerusalem. $\delta^{13}C$ and $\delta^{18}O$ values were calibrated relative to VPDB using one-point calibration with the internal Carrara Marble standard ($\delta^{13}C$ = -4.74, $\delta^{18}O$ = -3.85). The isotopic compositions reported here for internal standard represent long term averages calibrated to NBS-18 and NBS-19.

On the basis of the check standard, measurement precision was ±0.43 ‰ for $\delta^{13}C$ and ±0.46 ‰ for $\delta^{18}O$ (*df*=86). Measurement accuracy (systematic error) was evaluated by comparing the known and measured $\delta^{13}C$ and $\delta^{18}O$ values for Carrara Marble and factoring in the long-term uncertainty in these known measurements. Measurement bias due to systematic error (accuracy) was determined to be ±0.16 ‰ for both $\delta^{13}C$ and $\delta^{18}O$.

Mean and standard deviations of the measurements of the check standards for all analytical sessions containing data presented in this paper.

Session ID	Standard	n	$\delta^{13}C$ (‰, VPDB)	$\delta^{18}O$ (‰, VPDB)
Session 1	Carrara Marble	9	4.76 ± 0.07	-3.85 ± 0.07
Session 2	Carrara Marble	8	4.71 ± 0.19	-3.85 ± 0.11
Session 3	Carrara Marble	8	4.75 ± 0.23	-3.82 ± 0.12
Session 4	Carrara Marble	12	4.74 ± 0.07	-3.85 ± 0.14
Session 5	Carrara Marble	3	4.65 ± 0.16	-3.85 ± 0.17
Session 6	Carrara Marble	11	4.79 ± 0.19	-3.80 ± 0.20
Session 7	Carrara Marble	2	4.74 ± 0.10	-3.85 ± 0.20
Session 8	Carrara Marble	7	4.74 ± 0.13	-3.85 ± 0.16
Session 9	Carrara Marble	13	4.74 ± 0.28	-3.85 ± 0.24
Session 10	Carrara Marble	14	4.70 ± 0.16	-3.85 ± 0.14
Session 11	Carrara Marble	10	4.74 ± 0.26	-3.85 ± 0.12

Fourteen percent of the samples were analyzed in duplicate (33/230), the results of which are presented in Table S5. The measurement precision specific to the samples (the pooled standard deviation of all samples analyzed in duplicate) was ±0.54 ‰ for $\delta^{13}C$ and ±0.61 ‰ for $\delta^{18}O$ (*df*=33), following [70]. Standard uncertainty for $\delta^{13}C$ and $\delta^{18}O$ (u_c) was estimated following Szpak et al. (2017) and was determined to be ±0.46 ‰ for $\delta^{13}C$ and ±0.49 ‰ for $\delta^{18}O$.

Stable carbon and oxygen isotopic compositions for all samples analyzed in duplicate. "A" and "B" correspond to the analytical run in which the duplicates were measured.

Sample ID	Analytical run	$\delta^{13}C_A$	$\delta^{13}C_B$	$\delta^{18}O_A$	$\delta^{18}O_B$
HLZ27a-03	A=2, B=4	-10.07	-10.11	2.97	1.77
HLZ27a-05	A=2, B=4	-5.24	-4.41	3.33	3.12

HLZ27a-10	A=2, B=4	-5.50	-5.11	1.81	1.67
HLZ34b-07	A=3, B=4	-10.22	-10.32	3.64	2.57
HLZ35a-06	A=5, B=6	-3.23	-3.59	6.05	5.93
HLZ35b-12.1	A=6, B=6	-2.04	-3.54	5.64	4.09
HLZ35b-13.1	A=6, B=6	-4.56	-4.73	3.42	3.93
HLZ39b-09	A=1, B=2	-6.51	-8.55	3.15	4.67
HLZ42b-05	A=2, B=4	-8.47	-7.37	-0.54	-1.06
HLZ42b-06	A=2, B=2	-9.00	-8.09	0.30	0.28
HLZ42b-07	A=2, B=4	-9.80	-9.90	0.00	-0.87
HLZ42b-08	A=2, B=4	-10.19	-9.42	0.72	1.78
HLZ42b-09	A=2, B=4	-10.03	-9.76	2.27	2.46
NIZ04a-07	A=4, B=5	-7.54	-8.30	5.30	4.27
NIZ04b-12	A=5, B=6	-6.69	-6.51	3.60	5.18
NIZ08b-06	A=1,B=5	-9.07	-9.43	0.70	0.37
NIZ16a-03	A=1, B=2	-9.18	-10.41	3.14	2.32
NIZ16a-04	A=1, B=2	-10.57	-10.70	3.74	4.11
NIZ16a-06	A=2, B=4	-10.43	-10.87	5.23	5.10
NIZ16a-07	A=2, B=4	-10.51	-10.33	5.88	5.70
NIZ16a-08	A=2, B=4	-10.73	-11.63	6.91	6.08
NIZ16a-09	A=2, B=4	-10.89	-10.41	7.04	7.13
NIZ16a-10	A=2, B=4	-11.13	-10.09	8.22	7.06
SHV24a-07	A=2, B=4	-7.96	-6.78	1.56	1.10
SHV24a-09	A=2, B=4	-8.34	-7.92	3.18	3.87
SHV26b-02	A=1, B=4	-3.83	-3.79	3.97	5.20
SHV28a-04	A=3, B=5	-5.23	-5.89	5.57	3.72
SHV31a-03	A=2, B=4	-7.80	-6.81	4.32	4.35
SHV31a-04	A=2, B=4	-6.94	-6.93	6.04	5.38
SHV31a-06	A=2, B=4	-8.37	-8.25	5.92	5.42
SHV31a-07	A=4, B=5	-8.20	-7.88	4.47	4.92
SHV31a-10	A=4, B=5	-7.76	-8.18	2.29	1.97
SHV31a-13	A=2, B=4	-7.30	-7.38	0.76	0.09

Supplementary Data File S2 -- Calibration and Analytical Uncertainty for Tooth Dentine and Mandibular Collagen Isotopic Measurements ($\delta^{13}C$ and $\delta^{15}N$)

Stable carbon and nitrogen isotopic compositions were determined using an IsoPrime GVI Isotope Ratio Mass Spectrometer coupled to a Euro-Vector Elemental Analyzer at the Department of Biology at Boston University. $\delta^{13}C$ values were calibrated to VPDB and $\delta^{15}N$ values were calibrated to AIR using two-point calibration with the internal standards glycine and peptone (Table S6). The isotopic compositions reported here for internal standard represent long term averages calibrated to NBS-20, NBS-21, NBS-22 (for $\delta^{13}C$) and IAEA-N1, IAEA-N2, IAEA-N3 (for $\delta^{15}N$).

Standard reference materials used for calibration of $\delta^{13}C$ relative to VPDB and $\delta^{15}N$ relative to AIR.

Standard	Accepted $\delta^{13}C$ (‰, VPDB)	Accepted $\delta^{15}N$ (‰, AIR)
Glycine	+33.98	+10.68
Peptone	-14.75	+7.31

On the basis of the check standard, measurement precision was ±0.26 ‰ for $\delta^{13}C$ and ±0.47 ‰ for $\delta^{15}N$ (*df*=5). Measurement accuracy (systematic error) was evaluated by comparing the known and measured $\delta^{13}C$ and $\delta^{15}N$ for glycine and peptone and factoring in the long-term uncertainty in these known measurements. Measurement bias due to systematic error (accuracy) was determined to be ±0.10 ‰ for $\delta^{13}C$ and ±0.38 ‰ for $\delta^{15}N$. Standard uncertainty for $\delta^{13}C$ and $\delta^{15}N$ (u_c) was estimated following Szpak et al. (2017) and was determined to be ±0.27 ‰ for $\delta^{13}C$ and ±0.61 ‰ for $\delta^{15}N$.

Mean and standard deviation of all check standards for all analytical sessions containing data presented in this paper.

**Session ID**	**Standard**	**n**	**$\delta^{13}C$ (‰, VPDB)**	**$\delta^{15}N$ (‰, VPDB)**
Session 1	Glycine	5	-33.93 ± 0.08	10.78 ± 0.03
Session 1	Peptone	2	-14.77 ± 0.05	7.32 ± 0.07
